# The potential of 3rd‐generation nanopore sequencing for B‐cell clonotyping in lymphoproliferative disorders

**DOI:** 10.1002/jha2.815

**Published:** 2023-11-23

**Authors:** Marcus H. Hansen, Oriane Cédile, Niels Abildgaard, Charlotte G. Nyvold

**Affiliations:** ^1^ Haematology‐Pathology Research Laboratory, Research Unit of Haematology Department of Haematology, and Research Unit of Pathology Department of Pathology University of Southern Denmark and Odense University Hospital Odense Denmark; ^2^ OPEN, Odense Patient data Explorative Network, Odense University Hospital Odense Denmark

## Abstract

Lymphoid malignancies are characterized by clonal cell expansion, often identifiable by unique immunoglobulin rearrangements. Heavy (IGH) and light‐chain gene usage offers diagnostic insights and enables sensitive residual disease detection via next‐generation sequencing. With its adaptable throughput and variable read lengths, Oxford Nanopore thirdgeneration sequencing now holds promise for clonotyping. This study analyzed CD138+ plasma‐cell DNA from eight multiple myeloma patients, comparing clonotyping performance between Nanopore sequencing, Illumina MiSeq, and Ion Torrent S5. We demonstrated clonotype consistency across platforms through Smith‐Waterman local alignment of nanopore reads. The mean clonal percentage of IGH V and J gene usage in the CD138+ cells was 69% for Nanopore, 67% for S5, and 76% for MiSeq. When aligned with known clonotypes, clonal cells averaged a 91% similarity, exceeding 85%. In summary, Nanopore sequencing, with its capacity for generating millions of high‐quality reads, proves effective for detecting clonal IGH rearrangements. This versatile platform offers the potential for measuring residual disease down to a sensitivity level of 10^‐6^ at a lower cost, marking a significant advancement in clonotyping techniques.

Sensitive measurement of diagnostic markers in residual disease has played a long‐standing role in hematology. Besides the importance of quantifying low‐level transcripts or detecting somatic mutations, unique and clonally expanded rearranged immunoglobulin heavy‐chain (*IGH*) sequences can be identified in lymphoid malignancies at diagnosis by targeted next‐generation sequencing (NGS), even in difficult material such as formalin‐fixed, paraffin‐embedded tissue [1]. Importantly, NGS‐based methods have been shown to match clonotyping and minimal/measurable residual disease (MRD) detection by real‐time quantitative polymerase chain reaction (PCR) [2] or flow cytometry [3]. It is sensitive and specific, with validated reproducibility across laboratories, as shown by the EuroClonality‐NGS Working Group [4]. For more information, the topic has been extensively reviewed by Kotrova et al. [5].

Now, Oxford Nanopore (Oxford Nanopore Technologies (ONT), Oxford, UK) third‐generation sequencing is able to deliver a practical throughput of high quality and a broad range of read lengths. The versatility and applicability of nanopore sequencing are emphasized by its central role in the recent complete end‐to‐end mapping of the human genome [6], leading to *Method of the Year 2022* [7]. The platform has already proven valuable within research in hematologic malignancies, with emphasis on targeted sequencing [8]. Here, the study assessed whether the current state of Oxford Nanopore sequencing matches current standards in clonotyping B‐cell lymphoproliferative disorders.

We included CD138+ plasma‐cell DNA collected from eight patients with multiple myeloma. All the patient samples were thoroughly clonotyped and validated previously using Ion S5 and LymphoTrack *IGH* FR1 Assay (Invivoscribe, San Diego, CA) [9]. Nanopore library preparation on targeted *IGH* framework 1 amplicons (FR1, LymphoTrack) was performed using the Ligation Sequencing Kit (SQK‐LSK110) on 0.5 μg of pooled Illumina MiSeq (San Diego, CA, US) LymphoTrack amplicon DNA library. Following end‐repair, adapter ligation, and purification, the libraries were loaded onto primed flow cells (FLO‐MIN106D, ONT) and sequenced using MinION Mk1C (ONT).

The Nanopore MinION sequencing output counted 22.1 million reads (98%, EPI2ME, ONT) mapped to chromosome 14 and *IGH* locus (14q32.33). The average base calling accuracy was 89.8%. We discarded ligated sequences with multiple *IGH Variable* (*V*)*, Diversity* (*D*), and *Joining* (*J*) gene sequences (>800 bp), short or truncated sequences (<200 bp), and low‐quality reads (<Q20, see Supplement). 6.1 million reads (26%) fulfilled these criteria (*see data availability*), and 1.9 million (9%) contained intact patient‐specific indices for sample demultiplexing.

The median length of the nanopore reads was 488 bases (Figure 1A), corresponding to the size of rearranged *IGH V*(*D*)*J* genes flanked by adapter sequences, that is, identified Nanopore adapter [10], TruSeq Universal Adapter (Illumina), amplicon, and the index adapters (i7, Illumina). We implemented 64 vCPU multithreaded Smith‐Waterman (SW) alignment scoring for clonal identification.

**FIGURE 1:**
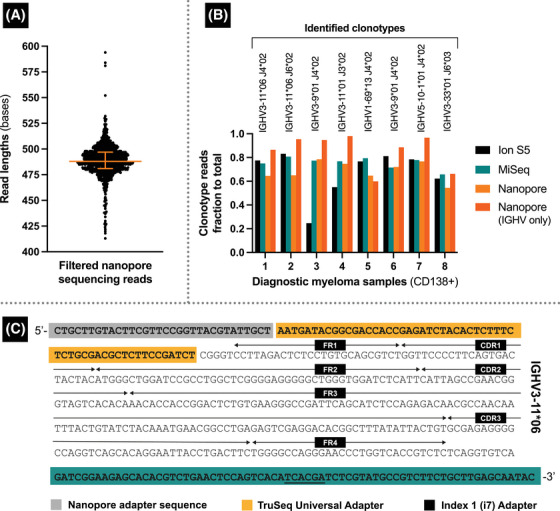
Targeted sequencing of the rearranged immunoglobulin heavy chain (*IGH*) locus using the Oxford Nanopore platform. The median length of the retained high‐quality reads (Q≥20, n = 6.1 × 10^6^) was 488 bp (A) with a high degree of concordance to MiSeq and Ion S5 in terms of identified clonotypes and clonotype burden (B). The general structure of the nanopore reads consisted of adapters and the *IGH*‐targeted amplicon (C, sequence from patient 1).

De‐novo clonotyping from local sequence alignments to IGMT *IGH V*/*J* genes [11] agreed with the results of LymphoTrack software based on MiSeq (5.9 million reads) and Ion S5 output (6.2 million reads). A minor exception was the assignment of polymorphism IGHV3‐11*06 versus IGHV3‐11*05 initially provided by the LymphoTrack software. However, complete concordance (8/8) was found when IgBLAST [12] or IMGT/V‐QUEST [11] labeled the *V*/*J* genes.

The identified monoclonal *IGH V*/*J* usage from the CD138+ cells constituted a mean clonal burden of 0.69 (0.54–0.79, Figure 1B) compared to 0.67 for Ion S5 and 0.76 for MiSeq. Including only the V‐gene yielded a higher fraction of 0.86 (0.6–0.98). When aligning the already identified clonotype sequence, the clonal plasma cell fractions constituted a fraction of 0.91 (0.71–0.97, > 85% similarity). This capacity to track specified sequences using nanopores is directly relevant for MRD detection, as discussed. A representative structure of the reads is exemplified in Figure 1C.

Although we show a complete capability of nanopore sequencing in identifying the clonotypes from multiple myeloma patient cases, this study is limited by the sole focus on de‐novo clonotyping from the clonally rearranged *IGH V*/*J* genes. Thus, our findings remain to be evaluated in frank MRD settings. However, it has recently been demonstrated in a preliminary and interesting study by Sampathi et al. that this is at least possible using cell‐free DNA from acute lymphoblastic leukemia [13].

We have previously estimated that the base‐calling error rate for Nanopore reads roughly 10 times higher than that of Illumina in a technically similar sequencing setup [14]. While the error rate constantly improves, correction may be performed [15], or high‐fidelity assays, such as duplex reads, can be implemented. Currently, the precision emphasizes the necessity for robust similarity scoring based on local sequence alignment. Here, this is performed using an adaptation of the versatile Smith‐Waterman algorithm. The apparent waste of reads, with more than 90% dispensed by filtering, is outweighed by precise identification of the clonal rearrangements in CD138+ plasma cells from patients with multiple myeloma.

Despite the limitations, the potential of running a sample per sequencing flow cell for sensitive MRD detection based on *IGH V*/*J* gene usage is directly apparent. In such a setup, the current MinION flow cell may provide sufficient coverage of several million reads to achieve a sensitivity level of 10^−6^, as long as adequate input DNA is supplied [16]. In our demonstration, we obtained more than 20 million reads for a single run, and the recent PromethION P2 Solo (ONT) promises to achieve much higher sample coverage. Furthermore, our preliminary findings cautiously suggest that the lower‐yield Oxford Nanopore Flongle flow cell, introduced in 2019, might offer a cost‐effective option for single‐use clonotyping of diagnostic samples. This hypothesis is based on its accessibility and the current gigabase sequencing output but requires further exploration.

In summary, nanopore sequencing demonstrates direct utilization in detecting clonal *IGH* rearrangements, comparable to Illumina and Ion Torrent in terms of *V*(*D*)*J* clonotyping and clonotype detection despite higher error rates. Current perspectives of this study include a low‐cost and versatile sequencing platform for measuring residual disease.

## AUTHOR CONTRIBUTIONS

MHH performed primary computational analyses on nanopore data and OC on Ion S5 and MiSeq. OC performed all laboratory work. CGN and NA provided internal funding, ensured ethical approvals, and selected samples for the project. MHH drafted the manuscript. All authors contributed to the study concept and the final version of the manuscript.

## CONFLICT OF INTEREST STATEMENT

The authors have no financial or nonfinancial interests to disclose.

## FUNDING INFORMATION

Haematology‐Pathology Research Laboratory, Research Unit of Haematology, Department of Haematology, Odense University Hospital; Brødrene Hartmanns Fond, Grant Number: A37628

## ETHICS STATEMENT

The project was approved by The Regional Committee on Health Research Ethics in Southern Denmark (S‐20160069), and data were handled in accordance with requirements by the Danish Data Protection.

## PATIENT CONSENT STATEMENT

A dispensation from written consent was obtained from the Regional Committee as excess material was collected from the diagnostic routine. No patient‐identifiable information is reported.

## CLINICAL TRIAL REGISTRATION

The authors have confirmed clinical trial registration is not needed for this submission.

## Supporting information

Supporting Information

## Data Availability

The quality‐filtered FASTQ sequencing data file is available for download at Figshare (Figshare LLC, Cambridge, MA, USA): https://doi.org/10.6084/m9.figshare.14207153.
