# Supplementary material for: The potential of 3rd‐generation nanopore sequencing for B‐cell clonotyping in lymphoproliferative disorders
Source: EJHaem. 2023 Nov 23;5(1):290–3. doi: 10.1002/jha2.815 (PMC10887334; doi:10.1002/jha2.815)
Supplement: Supplementary file 1 — Supporting Information [file JHA2-5-290-s001.pdf]

Supplement

The potential of 3rd-generation Nanopore sequencing for B-cell clonotyping in lymphoproliferative disorders

Marcus H. Hansen[1], Oriane Cédile[1,2], Niels Abildgaard[1], Charlotte G. Nyvold[1,2]
1) Haematology-Pathology Research Laboratory, Research Unit of Haematology, Department of Hematology, and Research Unit of Pathology, Department of Pathology, University of Southern Denmark and Odense University Hospital, Odense, Denmark
2) OPEN, Odense Patient data Explorative Network, Odense University Hospital, Odense, Denmark.

Analyses were performed on 64 vCPU cluster machine:

1. **Remove short and long reads (Q≥20)**

```
./q-filter 20 200 800 nanoporelymphotrack.fastq
```

2. **Score index adapter similarity in reads**

```
./swigh-score nanoporelymphotrack_filtered.fastq 63
GATCGGAAGAGCACACGTCTGAACTCCAGTCACATCTCGTATCCGTCTTCTGCTTG
```

3. **Remove sequences without index adapter sequences or low similarity (<75%)**

```
cat nanoporelymphotrack_filtered.fastq.out |cut -f1,2 -d' ' |awk '$2>0.75 {print}'|grep
"CAGTCAC[ATCG]\{6\}ATCTCGT" > nanoporelymphotrack_filtered.adapter.out
```

4. **Retrieve reads containing intact index**

```
index=ATCACG
p=1
cut -f1 nanoporelymphotrack_filtered.adapter.out |grep "CAGTCAC"$index"ATCTCGT" >
nanoporelymphotrack.patient$p.seq
```

5. **Retrieve reads containing intact index**

```
i=1
indices=(ATCACG TTAGGC TGACCA ACAGTG GCCAAT CAGATC ACTTGA)

for index in ${indices[@]}; do
    cut -f1 nanoporelymphotrack_filtered.adapter.out | grep "CAGTCAC"$index"ATCTCGT" > \
    nanoporelymphotrack.patient$i.seq
    ((i+=1))
done
```

6. **Dermine V gene or J for each sequence**

Flat files with V and J genes were downloaded from IMGT/V-Quest homepage in fasta format and converted to one line per gene.

```
./vgene-find nanoporelymphotrack.patient1.seq 63 [V or J gene flat file]
```

7. **Convert reads to fastq-like format**

```
cat nanoporelymphotrack.patient1.seq | awk '{print "@ID\n"$1"\n+\n"}' >
nanoporelymphotrack.patient1.pseudo.fastq
```

8. **Detect known clonotype sequence relevant for MRD detection**

```
./swigh-score nanoporelymphotrack.patient1.pseudo.fastq 63
GCGTCTGGATTCACCTTCAGTGACTACTACATGGGCTGGATCCGCCTGGCTCCAGGGAGGGGGCTG
GAGTGGATCTCATTCATTAGCCGAACGGGTAGTCACACAAACACCGCGGACTCTGTGAAGGGCCG
ATTCAGCATCTCCAGAGACAACGCCAACAATTTACTGTATCTACAAATGAACGGCCTGAGAGTCGA
GGACACGGCTTTATATTACTGTGCGAGAGGGGGCCAGGTCAACTGGGAATTACCTGACTTCTGGGG
CCAGGGAACCCT
```

9. **Count sequences with more than 85% similarity to clonotype**

```
cat nanoporelymphotrack.patient1.pseudo.fastq.out | awk '$2>0.85 {print $3}' | paste -s -d+ - |bc
```

C code for quality filtering of fastq file (file: q-filter.c)

```
#include <stdio.h>
#include <string.h>
#include <math.h>
#include <stdlib.h>

int main(int argc,char* argv[]) {

	int lowerlength = 200;
	int upperlength = 800;
	int lowerqual = 20;


	FILE *fp;
	int len = 1000000;
	char buff[len], ch, qual[len], dna[len], plus[len], id[len];
	int lines = 0;
	fp = fopen(argv[1], "r");
	int i = 0;
	int u;
	double score = 0.00;
	int mean;

	while(!feof(fp)) {
		ch = fgetc(fp);
		if(ch == '\n') {
			lines++;
		}
	}

	printf("%i",lines);
	rewind(fp);

	while (i <= lines/4) {
		i++;
		fgets(buff, len, fp);
		strcpy(id, buff);
		fgets(buff, len, fp);
		strcpy(dna, buff);
		fgets(buff, len, fp);
		strcpy(plus, buff);
		fgets(buff, len, fp);
		strcpy(qual, buff);
		score = 0;
		for (u = 0; u < strlen(buff); u++) {
			score = score + (buff[u]-33);
		}

		mean = (score/strlen(buff));
		if(mean>=lowerqual && strlen(dna)>=lowerlength && strlen(dna)<=upperlength) {
			printf("%s", id);
			printf("%s", dna);
			printf("%s", plus);
			printf("%s", qual);
		}
	}
	fclose(fp);
	return 0;
}
```

Linux wrapper for Smith-Waterman scoring (file: swigh-score)

```
#!/bin/bash


mkdir tmp

echo "Retrieving sequences of lengths above 200 bases for local Smith-Waterman alignment"
awk 'NR%4==2 {a=$2; if(length($a)>1) print $a}' $1 | sort | uniq -c | tr -s " " "\t" |
awk '{ print $2"\t"$1}' | sort > tmp/counts.txt
cut -f1 tmp/counts.txt > tmp/stack.fastq

n=$(< tmp/counts.txt wc -l)
lines=$(awk -v num=$n -v threads=$2 'BEGIN {print int(num/threads)}')

echo "Splitting fastq file into chunks of $lines lines"
split -l $lines tmp/stack.fastq tmp/split.stack.

Files="split.stack.*"

for f in 'tmp/'$Files
do
	echo "Performing local alignment of " $f
	bin/SWscoresequences.sh $f $3 > $f.out.txt &
done

wait

echo "concatenating output..."
cat tmp/split.stack.*.out.txt | sort > tmp/$1".SWscore.out"
join tmp/$1".SWscore.out" tmp/counts.txt | sort -k2nr > $4$1".out"

rm -r tmp
```

Script for calling smith-Waterman scoring (file: bin/SWscoresequences.sh)

```
#!/bin/bash

while read p; do
  bin/SWscore "$p" $2
done < $1
```

C code for the identification of V and J genes (SWscore)

```
#include<stdio.h>
#include<string.h>


int main(int argc,char* argv[])
{
	int match = 0;
	double val = 0.000;
	int cnum1 = strlen(argv[1]);
	char str1[cnum1];
	int cnum2 = strlen(argv[2]);
	char str2[cnum2];
	double SWpercent;

	strcpy(str1,argv[1]);
	strcpy(str2,argv[2]);

	int arr[cnum2][cnum1];

	for(int i=0;i<cnum2;i++){
		for(int j=0;j<cnum1;j++){
			if(str1[j]==str2[i])
```

```
                    arr[i][j]=1;
                else
                    arr[i][j]=0;
            }
        }

        int c;
        int ul;
        int l;
        int u;

        for(int i=1;i<cnum2;i++){
            for(int j=1;j<cnum1;j++){

                c=arr[i][j];
                ul=arr[i-1][j-1];
                l=arr[i][j-1];
                u=arr[i-1][j];


                if( c>0 && (ul+1)>(l-1) && (ul+1)>(u-1)) {
                    arr[i][j] = ul+1;

                } else if( c==0 && (ul+1)>(l-1) && (ul+1)>(u-1)) {
                    arr[i][j] = ul-1;

                } else if((l-1)>(u-1)) {
                    arr[i][j] = l-1;

                } else if((u-1)>(l-1)) {
                    arr[i][j] = u-1;
                } else {
                    arr[i][j] = 0;
                }


                if(arr[i][j]<0) arr[i][j] = 0;

                if(arr[i][j]>val) val = arr[i][j];

            }
        }

        if(cnum1 < cnum2)
            SWpercent = val/(double)cnum1;
        else
            SWpercent = val/(double)cnum2;

        printf("%s\t", str1);
        printf("%f\n", SWpercent);

        return 0;
}
```

Shell wrapper for detetmining V or J gene (file: vgene-find)

```
#!/bin/bash

mkdir tmp

echo "Subsampling 1000 sequences for clonotyping"
head -n 1000 $1 > tmp/seq.txt

n=$(< tmp/seq.txt wc -l)
lines=$(awk -v num=$n -v threads=$2 'BEGIN {print int(num/threads)}')

echo "Splitting fastq file into chunks of $lines lines"
split -l $lines tmp/seq.txt tmp/split.seq.stack.
```

```
Files="split.seq.stack.*"

for f in 'tmp/'$Files
do
     echo "Performing local alignment of " $f
     ./vj $f $3 > $f.out.txt &
done

wait

echo "concatenating output..."
cat tmp/split.seq.stack*.out.txt > tmp/igv.out

cat tmp/igv.out |awk '$1>0.01 {print $2}' | sort | uniq -c | awk '{print $1"\t"$2}' |
sort -k1nr

rm -r tmp
```

C code for the identification of V and J genes (file: vj)

```
#include<stdio.h>
#include<string.h>


float SW(char* arg1, char* arg2) {

     int match = 0;
     double val = 0.000;
     int cnum1 = strlen(arg1);
     char str1[cnum1];
     int cnum2 = strlen(arg2);
     char str2[cnum2];
     double SWpercent;

     strcpy(str1,arg1);
     strcpy(str2,arg2);

     int arr[cnum2][cnum1];

     for(int i=0;i<cnum2;i++){
          for(int j=0;j<cnum1;j++){
               if(str1[j]==str2[i])
                    arr[i][j]=1;
               else
                    arr[i][j]=0;
          }
     }

     int c;
     int ul;
     int l;
     int u;

     for(int i=1;i<cnum2;i++){
          for(int j=1;j<cnum1;j++){

               c=arr[i][j];
               ul=arr[i-1][j-1];
               l=arr[i][j-1];
               u=arr[i-1][j];


               if( c>0 && (ul+1)>(l-1) && (ul+1)>(u-1)) {
                    arr[i][j] = ul+1;

               } else if( c==0 && (ul+1)>(l-1) && (ul+1)>(u-1)) {
                    arr[i][j] = ul-1;
```

```
            } else if((l-1)>(u-1)) {
                 arr[i][j] = l-1;

            } else if((u-1)>(l-1)) {
                 arr[i][j] = u-1;
            } else {
                 arr[i][j] = 0;
            }


            if(arr[i][j]<0) arr[i][j] = 0;

            if(arr[i][j]>val) val = arr[i][j];

        }
    }

    if(cnum1 < cnum2)
        SWpercent = val/(double)cnum1;
    else
        SWpercent = val/(double)cnum2;

    return SWpercent;
}

int main(int argc,char* argv[]) {

    FILE *fp,*fp2;
    int len = 100000;
    char ch, ch2,buff[len],dna[len],dna2[len],sw[len],sw2[len];
    int lines = 0;
    int lines2 = 0;
    fp = fopen(argv[2], "r");
    fp2 = fopen(argv[1], "r");
    int i = 0;
    int i2 =0;
    float f=0.0000;
    float score=0.0000;
    while(!feof(fp)) {
        ch = fgetc(fp);
        if(ch == '\n') {
            lines++;
        }
    }

    while(!feof(fp2)) {
            ch2 = fgetc(fp2);
            if(ch2 == '\n') {
                    lines2++;
            }
      }

    rewind(fp);
    rewind(fp2);
    while (i2 <= lines2) {
        i2++;
        fgets(buff, len, fp2);
        strcpy(dna2, buff);
        while (i <= lines) {
            i++;
            fgets(buff, len, fp);
            strcpy(dna, buff);
            f=SW(dna2,dna);
            if(f>score) {
                score=f;
                strncpy(sw, dna, strlen(dna)-1);
                strncpy(sw2, dna2, strlen(dna2)-1);
            }
```

```
            }
            if(score<0.9999) {
                 printf("%f\t%s\t%s\n", score, sw, sw2);
            }
            rewind(fp);
            score=0.0000;
            i=0;
      }
        return 0;
}
```

Clonotype sequences

```
> Identified patient 1 clonotype sequence
GCGTCTGGATTCACCTTCAGTGACTACTACATGGGCTGGATCCGCCTGGCTCCAGGGAGGGGGCTGGAGTGGATCTCATTCATTAGCCG
AACGGGTAGTCACACAAACACCGCGGACTCTGTGAAGGGCCGATTCAGCATCTCCAGAGACAACGCCAACAATTTACTGTATCTACAAA
TGAACGGCCTGAGAGTCGAGGACACGGCTTTATATTACTGTGCGAGAGGGGGCCAGGTCAACTGGGAATTACCTGACTTCTGGGGCCAG
GGAACCCT

> Identified patient 2 clonotype sequence
GCCTCTGGATTCACCTTCAGTGACCACTACATGAGCTGGATCCGCCAGGTTCCAGGGAAGGGGCTGGAGTGGGTTTCATACATTAGTAG
TGGTGGTAGTTACACAAACTACGTAGACTCTGTGAGGGGCCGATTCACCATTTCCAGAGACAACGCCAAGAAGTCAATGTATCTGCACA
TGAGCAGCCTGAGAGGCGAGGACACGGCTGTCTATTACTGTGCGAGAGTTATGAGGCACTACGATTCCTACGGAATGGACGTCTGGGGC
CAAGGGACCAC

> Identified patient 3 clonotype sequence
GTCTCTGGATTCAGTTTTCATGATTATGGCATGCACTGGGTCCGGCAAGCTCCAGGCAAGGGCCTGGAGTGGGTCTCAGGTATTCATGA
CAAGAGTAATTCCATAGGCTATGCGGACTCTGTGAAGGGCCGATTCACCATCTCCAGAGACAACGCCAAGAACTCCCTCCTTCTGCAAA
TGAACAGTCTGAGACCTGAGGACACGGCCTTGTATTACTGTGTAAAAGATTTTTCGAATCACTTTGATAGTCTTGGTTATCTTGACTCC
TGGGGCCAGGGCACCCT

> Identified patient 4 clonotype sequence
GTCTCTGGATTCAGTTTTCATGATTATGGCATGCACTGGGTCCGGCAAGCTCCAGGCAAGGGCCTGGAGTGGGTCTCAGGTATTCATGA
CAAGAGTAATTCCATAGGCTATGCGGACTCTGTGAAGGGCCGATTCACCATCTCCAGAGACAACGCCAAGAACTCCCTCCTTCTGCAAA
TGAACAGTCTGAGACCTGAGGACACGGCCTTGTATTACTGTGTAAAAGATTTTTCGAATCACTTTGATAGTCTTGGTTATCTTGACTCC
TGGGGCCAGGGCACCCT

> Identified patient 5 clonotype sequence
CTTCTGGAGGCACCTTCAGCAAATATGCTATCAGTTGGTTGCGACAGGCCCCTGGACAAGGGCTTGAGTGGATGGGAGGGACCATCGCT
ATCTTTGGTGCTACAAACTACGCACAGAAGTTCCAGGGCAGAGTCACGATTACCGCGGACGAATCCACGAGCACTGCCTACATGGAGTT
GAGCAGCCTGACATCTGAGGACACGGCCGTATATTATTGTGCGAGAGGTAGTAGTGGCTTCTTTGACTACTGGGGCCAGGGAACCCT

> Identified patient 6 clonotype sequence
GCCTCTGGATTCACCTTTGATGATTATGCGATGCACTGGGTCCGACAAGTTCCAGGGAAGGGCCTGGAGTGGGTCTCAGGAATTAGCTG
GAATGGTGGTTCCAGAGGCTATGTGGACTCTGTGAAGGGCCGATTCACCATCTCCAGAGACAGTGCCAAGAATTCCCTCTCTCTGCAAA
TGAACAGTCTGAGAACTGAGGACACGGCCCTCTATTACTGTGTCAAAGCTCGAGGGTATAACAGTGGCTGGTTCGAGTCCCCGTTTGAG
TCCTGGGGCCAGGGCACCCT

> Identified patient 7 clonotype sequence
GCTCTGAATACAGCTTTACCAAGTACTGGATCAGCTGGGTGCGCCAGATGCCCGGGAAGGGCCTGGAGTGGATGGGGAGGATTGATCCC
AGTGACTCTTATACCTACTACAGCCCGTCCTTCCAAGGCCACGTCACCATCTCAGCTGACAAGTCCATCACCACTGCCTATCTGGAATG
GAGCAGCCTGAAGGCCTCGGACACCGCCATGTATTACTGTGCGAGACGGTCCAATTGTCGTGATACCAATTGCTTTACGTGGAACCCCT
ACTATTTTGACTCTTGGGGCCAGGGAACCCT

> Identified patient 8 clonotype sequence
GCGTCTGGATTCACCTTCAGTAATTACGGCATGCACTGGGTCCGCCAGGCTCCAGGCAAGGGGCTGGAGTGGGTGGCAGTCATATGGTT
TGATGGCAGCGATACATATCATGCAGACTCCGTGAAGGGCCGGTTCACCATTTCCAGAGACAATTCCATCAATACGCTTTATCTGCAAA
TGAACAGCCTGAGAGCCGAGGACACGGCTGTGTATTACTGTGCGAGAGACCCACCCCGTGAATATAGTGGCTACTACCAATACATGGAC
GTCTGGGGCAAAGGGACCAC
```
